# Sustainable conversion of biomass to rationally designed lithium-ion battery graphite

**DOI:** 10.1038/s41598-022-11853-x

**Published:** 2022-05-16

**Authors:** Nathan A. Banek, Kevin R. McKenzie, Dustin T. Abele, Michael J. Wagner

**Affiliations:** Department of Chemistry, George Washington University, Washington, DC USA

**Keywords:** Materials for energy and catalysis, Batteries, Climate-change mitigation, Sustainability, Energy, Solid-state chemistry, Carbon capture and storage, Batteries

## Abstract

The carbon net negative conversion of bio-char, the low value byproduct of pyrolysis bio-oil production from biomass, to high value, very high purity, highly crystalline flake graphite agglomerates with rationally designed shape and size tailored for lithium-ion battery energy storage material is reported. The process is highly efficient, 0.41 g/Wh; the energy content of its co-product of the process, bio-oil, exceeds that needed to power the process. It is shown that the shape of the starting material is retained during the transformation, allowing the ultimate morphology of the graphite agglomerates to be engineered from relatively malleable biomass. In contrast to commercial graphite production, the process can be performed at small scale with low equipment costs, enabling individual research laboratories to produce Li-ion grade graphite with customizable shape, size and porosity for Si/graphite composite and other graphite involved anodes. The mechanism of the graphitization of bio-char, a “non-graphitizable” carbon, is explored, suggesting the molten metal catalyst is absorbed into the pore structure, transported through and transforming the largely immobile biochar. Finally, the transformation of biomass to rationally designed graphite morphologies with Li-ion anode performance that closely mimic commercial shaped graphite is demonstrated.

## Introduction

Combating global climate change will require vast utilization of bioenergy and carbon negative or neutral “green” materials production and utilization^1^. It has been proposed that these processes should be made economically competitive by carbon valuation because finding market competitive solutions to replace carbon pricing (e.g. carbon tax or emission permits) is extremely challenging. Lignocellulose pyrolysis is a particularly attractive biomass conversion process, providing sustainable and carbon–neutral electricity, liquid biofuel and chemical feedstocks^2^. A growing number of large scale biomass pyrolysis plants have come online in recent years including a full scale combined heat and power plant (district heat, 210 GWh electricity, and 50,000 ton/year bio-oil) in Joensuu, Finland. Biomass pyrolysis oil is the least expensive carbon neutral replacement for transportation fuels, which account for 27% of all greenhouse emissions, but even so, it is not market competitive with fossil fuels. Attaining market viability depends on driving down the cost of bio-oil by upgrading or valorization of its generally burned waste product, biochar, to value-added products^3^.

Graphite is an attractive target valorization product of biochar. Graphite is classified as a “strategic and critical mineral” by the US and EU, with a market that is expected to nearly double to reach $28.33 billion by 2026^4^. While graphite is used in numerous applications, its market growth is expected to be driven by the increasing demand for Li-ion grade graphite, with Li-ion battery “mega-factories” being built to supply the needs of electric vehicles (EV). Meeting the graphite needs to limit global temperature rise to 2 °C will require a 500% increase in production by 2050 according to a recently published report from The World Bank^5^. However, severe supply shortages are predicted^6,7^, with potentially negative impacts to EV production and increased costs. Thus, conversion of biochar to Li-ion grade graphite could help meet the needs of a very large and growing market, while increasing its value by ~ 1000 fold^8^, and enabling the material needs for the wide adoption of zero emission EV’s.

Current graphite production, whether obtained through high temperature (3000 °C) transformation (synthetic graphite) of highly pure graphitizable carbons or mining (natural graphite), is highly deleterious to the environment. Synthetic graphite production is highly energy intensive (~ 7500 kWh/t)^9^ and results in large greenhouse gas emissions^10^. Mining is devastating to the landscape and purification of natural graphite requires large-scale use of environmentally harmful agents such as HF and H_2_SO_4_^11^. Micronizing and shaping (“rounding”) the graphite, necessary for Li-ion battery application, results in significant (~ 70%) material loss. Coating and “re-graphitizing” adds further environmental impact and cost. While the academic researchers actively participated in the development and improvement of graphite anodes for Li-ion batteries, their contributions have dwindled in the past decade due to the complexity and facility requirements of conventional graphitizing and processing to produce graphite appropriate for Li-ion batteries, limiting research primarily to modifications, applications and properties of systems based on commercial graphite.

Graphite is entrenched as the predominant anode active material in commercial Li-ion batteries, and is likely to remain so for the foreseeable future despite intense research efforts over the past two decades to find a higher energy density replacement. Most recently, Si has been of particular interest as a replacement for graphite as an anode material, due to its extremely high gravimetric and volumetric capacities, almost ten times higher than those of graphite, low working potential and abundance. However, development has been challenging due to the very large expansion of Si upon lithiation, up to ~ 300%, which leads to electrode structural degradation during cycling, ineffective passivation and low Coulombic efficiency (CE). Even so, electric vehicle (EV) battery manufacturers have successfully incorporated small percentages Si materials, generally SiO_x_, as capacity boosting additives to graphite anodes in commercial cells. It has been proposed that practical incorporation of Si into Li-ion cells requires a similar strategy, that is, being blended into composites with graphite^12^. While numerous investigations of composites of Si and Li-ion graphite have been reported recently, they have employed commercial Li-ion graphites that were tailored by industry to achieve excellent performance as the sole or primary active material, including high packing density and thus volumetric capacity, rather than optimal properties, including shape and porosity, to host Si^13–22^. As was pointed out recently, development of Si/graphite composites requires that “both the graphite and Si parts should be engineered”^12^, a task that is greatly encumbered by the need to use commercial graphite as a starting material for modification and blending, rather than synthesizing customized graphite.

Recently, it was first reported that biochar and other non-graphitizable carbons could be converted to high purity (99.95% C), highly crystalline graphite (biochar graphite or BCG) at a laboratory scale with equipment requirements readily within the financial means of many academic laboratories^8^. It was shown that biomass could be readily converted to “potato” graphite, morphologically similar to some commercial graphites, however, its relatively high surface area (10.3 m^2^/g) resulted in low initial CE, 84%, unacceptably below that of commercial Li-ion anode graphite (~ 90%). While the control of graphite flake size, a critical parameter for Li-ion battery performance, was demonstrated, the ability to rationally select the agglomerate shape and size was not, nor was an understanding of the origin of the morphology obtained presented. Additional reports have appeared demonstrating the conversion of biomass to anode materials with varying degrees of graphitic character and battery performance, but without rational morphology control^23–28^.

In an effort to gain mechanistic insight into the BCG graphitization process, the effects of process parameters on yield and agglomerate morphology were studied and are presented herein. It is shown that the size and shape of the graphite agglomerates can be rationally controlled to a remarkable degree. Finally, the rational synthesis of graphite with a predetermined flake size, and agglomerate size and shape, is demonstrated and shown to closely compare to commercial Li-ion graphite. The methods described will allow researchers to produce graphite at a laboratory scale with tailored morphological properties, including porosity, to tailor its performance as anode active material and/or alloying material host, with the potential to scale to industrial quantities, without the environmental impact of current graphite production.

## Results and discussion

### Power and wavelength dependence

**Figure 1:**
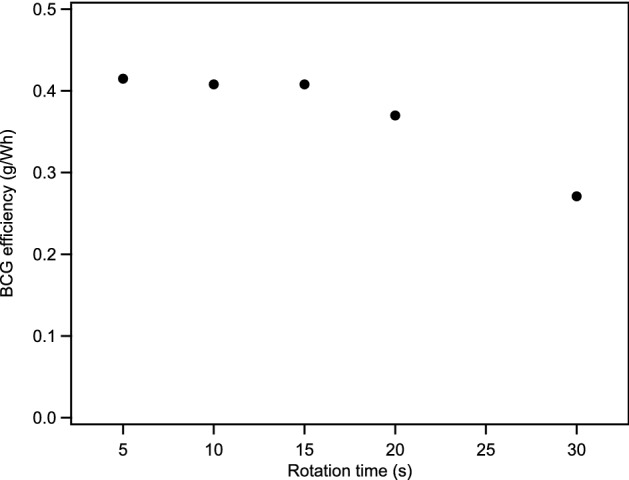
Graphite production irradiated energy efficiency plotted as a function of the duration of a full rotation of the pellet while irradiated at 200 W.

The previously published biomass char to graphite conversion results were obtained with a 60 W CO_2_ laser (10.6 µm) beam irradiating the sample during a single 48 s rotation resulting in a graphite production irradiated energy efficiency (termed “BCG efficiency” herein) of 0.25 g/Wh^8^. For this study, a 200 W diode laser (980 nm) was used instead of a CO_2_ laser. The effect of total irradiation power on BCG efficiency was studied by irradiating the biochar/Fe pellets at 200 W during one complete rotation at a variety of rotation times (Fig. 1). It should be noted that the actual irradiation time of any particular part of the pellet is extremely short; the laser is focused to a 2 mm diameter spot on the pellet, thus the irradiation duration is 0.159 s when the full pellet rotation time is 5 s. Increasing the irradiation time from 5 to 15 s has no effect on the BCG efficiency (0.41 g/Wh, 2400 kWh/t), with essentially 100% of the biochar carbon on exterior of the pellet being converted to graphite, accounting for more than 75% of the entire biochar, forming a thick brittle layer that can easily be removed by gentle abrasion from the unconverted interior biochar. Further increases in irradiation time resulted in diminishing BCG efficiency as the remaining unconverted biomass was shielded from the laser by the exterior. However, while slightly less energy efficient, more that 90% of the biochar was converted to graphite with a 20 s irradiation time with full conversion being achieved by ~ 30 s. The total energy input for biochar conversion with maximum BCG efficiency, 0.83 Wh (200 W for 15 s), is nearly identical to that input by a CO_2_ laser in the previous study, but the efficiency is 64% higher. This result is indicative of a power and/or laser wavelength dependence on the BCG efficiency obtained.

**Figure 2:**
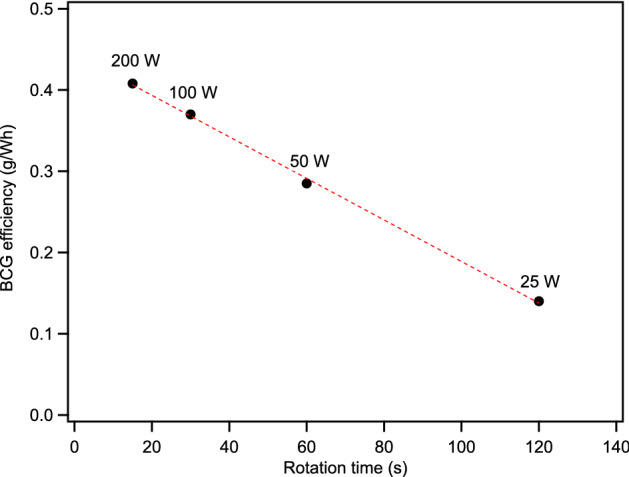
Graphite production irradiated energy efficiency plotted as a function of the duration of a full rotation of the pellet while irradiated at indicated laser output power scaled for a constant energy input of 0.83 Wh. Dashed line is a linear least squares fit of the data.

The effect of power was examined by varying the laser output power and rotation speed to keep the total energy input constant at 0.83 Wh of laser irradiation. The BCG efficiency for each power setting and revolution time increased linearly with power despite the cumulative energy input remaining constant (Fig. 2), indicating strong power dependence. Extrapolation of a linear least squares fit of the data indicates graphite is not produced at energy inputs of less than 17.2 W (173.8 s rotation time). Efforts to make BCG at 15 W with a 240 s rotation time (1.0 Wh) resulted in a low yield (< 0.05 g/W) of poorly graphitized material (Fig S2), with a very small number of seemingly well-graphitized flakes located during extensive examination by SEM (Fig S3).

The effect of the wavelength yield is significant, but much less than that of the power. The production rate at 50 W (0.285 g/Wh) exceeded our previous results with the 60 W CO_2_ laser (0.25 g/Wh) at 0.83 Wh of laser irradiation. The wavelength dependence may be due to the increase in absorption of the Fe metal and the biochar as the irradiation wavelength is lowered from 10.6 µm to 980 nm, resulting in more efficient energy transfer to the sample.

### Effect of biomass processing on BCG morphology

**Figure 3:**
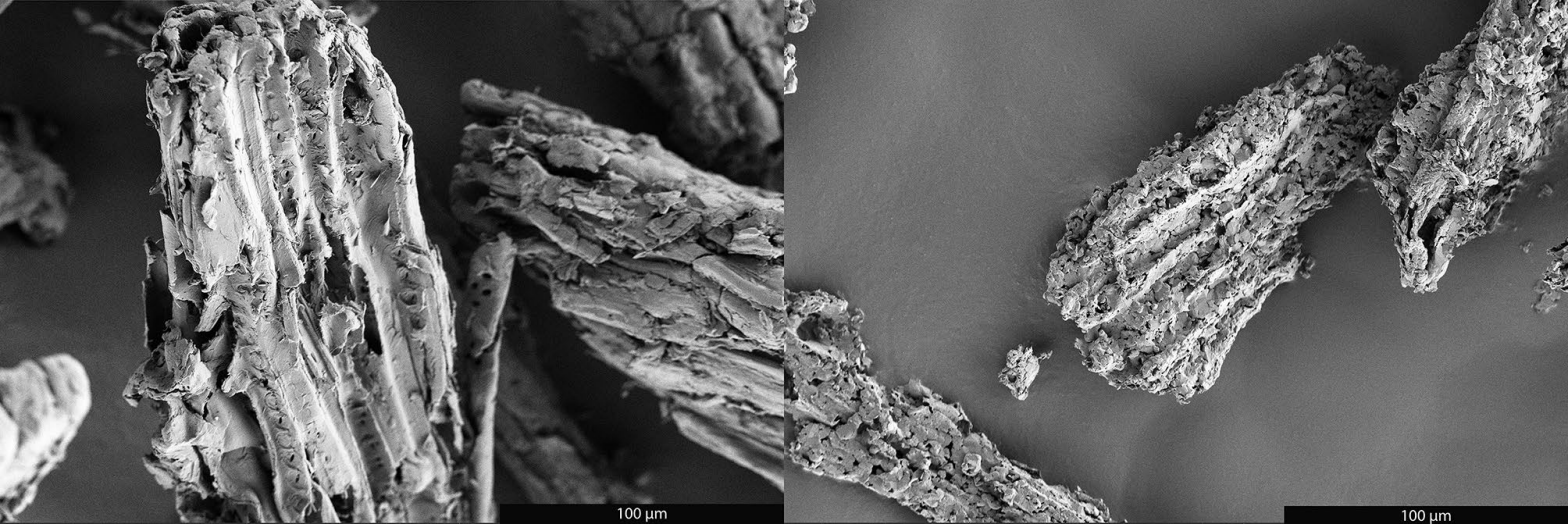
SEM images of sawdust char (left) and BCG made from sawdust char (right) showing preservation of wood-like morphology during graphitization.

The previous study demonstrated that the BCG flake size was determined by the size of the Fe metal catalyst particles used, however, what determines the morphology of the BCG was left unclear. Why were “potato” shaped agglomerates obtained under some conditions and simple flakes under others? Mixing of the Fe and biomass in the previous study was achieved by ball milling that can result in significant changes to the biomass morphology that may, in turn, affect the morphology of the BCG produced. To investigate the effects of milling on morphology, samples milled for 30 min, as was the case in the previous study, were compared to those milled for 1 min. The morphology of the resulting BCG, produced with essentially identical yields, is dramatically different. Reducing the milling time to 1 min resulted in BCG that is of remarkably similar morphology to the starting biomass (Fig. 3). Intricate morphological details of the sawdust are preserved upon graphitization. Examination at higher magnification shows that the graphite platelets on the exterior of the agglomerates are oriented with their basal planes facing the agglomerate exterior (Fig S4). In contrast, BCG from biomass samples milled for 30 min consists of agglomerates of graphite flakes, similar to commercial “potato” or “shaped” graphite (Fig S5). Thus, it appears that the formation of “potato” shaped graphite agglomerates is due to physical alteration of the starting biomass during milling. It should be noted that milling the biomass for 30 min to form “potatoes” reduces the surface area of the resulting graphite from 11 to 7.5 m^2^/g and increases its density, attributes that are favorable for Li-ion battery anode application.

### Graphitization of spherical algae

**Figure 4:**
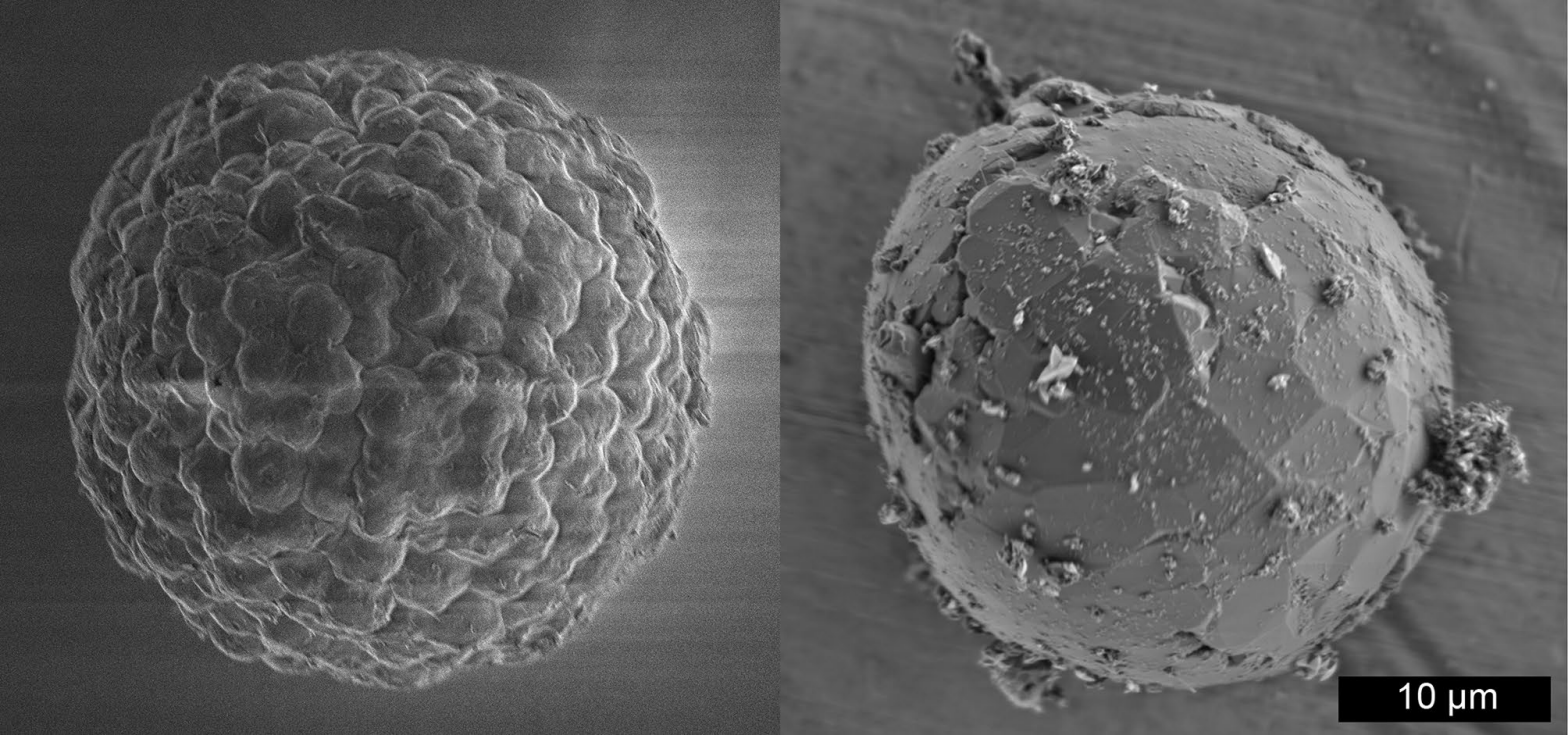
SEM images of raw spherical algae (left) and BCG made from spherical algae (right). Note that the BCG surface appears to be composed of the basal planes of interconnected graphite flakes.

To further investigate the morphological retention of the biomass upon graphitization, the BCG process was used to graphitize dried chlorella (spherical) algae (Fig. 4). As received, the algae are roughly spherical with a textured surface reminiscent of a raspberry. The spherical shape of the algae was retained upon graphitization, with the irregular surface of the raw algae transformed to graphite with the basal planes of interconnected graphite crystallites forming a continuous surface. The algae char derived BCG consists of fragile, empty shells composed of very thin (< 1 µm) graphite walls. Most of the shells are broken during processing, possibly the result of grinding during purification (Fig S6).

### BCG formation mechanism

It was previously shown that the metal catalyst is melted during the BCG process but is not macroscopically transported^8^. Further, simple dissolution of the carbon into the catalyst and precipitation as graphite is unlikely to explain the bulk of the process as the quantity of carbon converted is at least five times larger than its solubility in the catalyst. It was proposed that the BCG process initially proceeds by a mechanism analogous to that by which hollow carbon nanospheres are formed, that is, by the formation of carbon shells from graphene-like conjugated macromolecules freed by the disruption of the cross-linking of the biochar microdomains^8^. Initial shell formation occurs first at the catalyst surface with the formation of subsequent shells restricting the interior space. The exothermic crystallization of these turbostratically ordered graphene shells compresses and heats the molten catalyst that eventually fractures the shells. The remnants of the shells then relax into the thermodynamically favored planar structure, resulting in graphite crystals with basal dimensions that are approximately the diameter of the catalyst. Freed from the confines of the graphene shells, the catalyst can then move into untransformed biochar to repeat the process.

**Figure 5:**
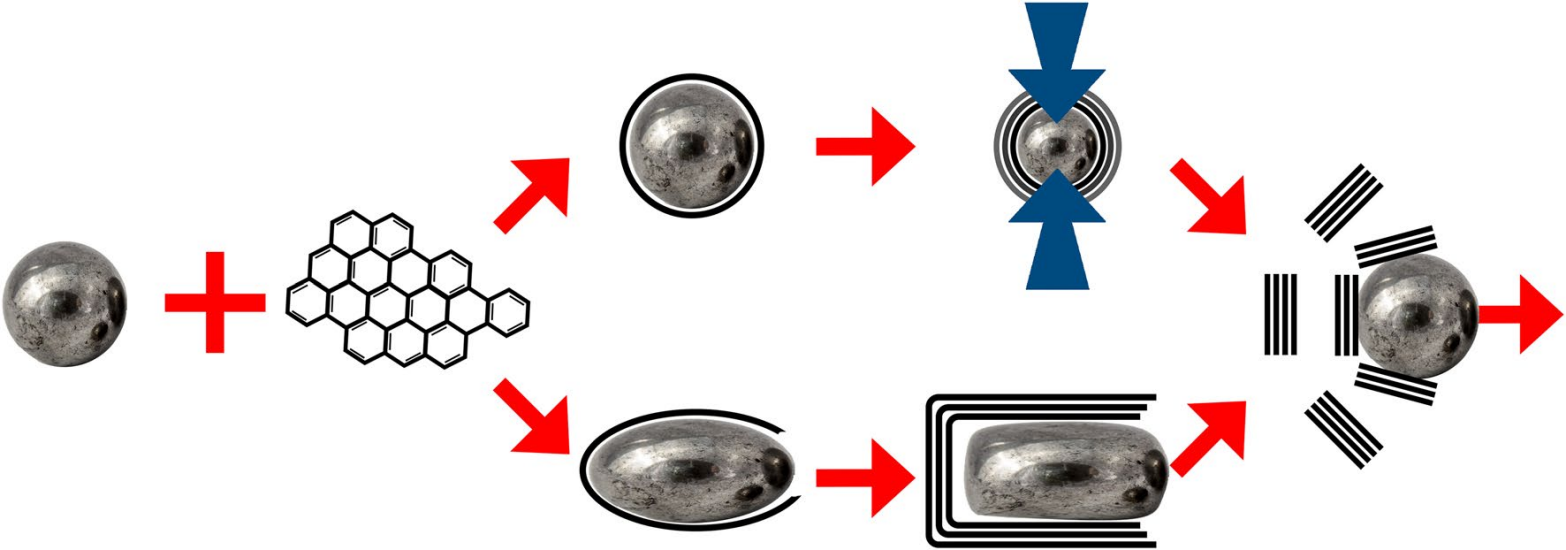
Two possible BCG graphitization paths.

Here it is shown that the biochar is remarkably stationary, with the biomass particulate structure retained with a high degree of detail. The complete conversion to graphite observed and the immobility of the biochar indicates that the catalyst enters the biochar and migrates through it. Catalyst transport along grain boundaries would not be sufficient, as it would only result in graphitization of the surfaces of the biochar particles. As illustrated in upper path in Fig. 5, following initial formation of graphene shells and pressure buildup, the shells rupture toward the exterior of the biochar, away from the solid mass and toward the grain boundary, resulting in surface graphite that is preferentially oriented with its basal plane to the exterior, as experimentally observed, and the expulsion of the catalyst into the biochar for further graphitization. However, it should be noted that the development of complete shells around the catalysts might not be necessary or occur. Graphene walls that break off to becomes planer graphene flakes might only form at the side and rear of the Fe as it moves through the biochar, with continuous addition of carbonaceous species from the front of the catalyst, in a manner similar to the growth of carbon nanotubes, and illustrated in the lower path of Fig. 5^29^.

The BCG process is only enabled with sufficient energy input to rapidly heat the catalyst; slower heating has been shown to result in graphene shell formation but not graphite previously^30^ and confirmed here. Slower heating may result in formation of shells prior to transport of the catalyst into the biochar, blocking access and thus preventing graphitization. With rapid heating, a large concentration of highly conjugated graphene macromolecular precursors can be incorporated into the molten catalyst prior to the formation of the first shell that prevents further carbon addition. The high concentration may be required to form a sufficient number of graphene shells to provide the pressure necessary for rupture rather than stable graphene shell formation.

The entry of the catalyst into the biochar could be initiated by adsorption and absorption of carbon species in abutting particles, eroding the biochar from the surface, however, it seems likely that such a process would proceed simultaneously from multiple biochar particles with wall formation and disruption preventing the migration of the catalyst into the biochar and not preserve biomass morphology. It seems more likely that the entry of the catalyst into the biochar is, at least in part, driven by capillary action. This explanation is consistent with the observation that while the BCG process efficiently graphitizes “non-graphitizable” porous carbons such as biomass and lower ranked coal (lignite) chars, attempts to graphitize fusing bituminous coal, a “graphitizable” carbon that forms a char with an inaccessible pore structure, have failed. Intrusion of the molten catalyst into the pore structure and absorption of the carbonaceous species could connect multiple pores to provide a continuous space in which graphitization occurs. Capillary action could contribute to the continued percolation of the catalyst through the biochar, allowing graphitization to proceed with morphology retention.

### Rational design of Li-ion anode graphite—simple size selection

The understanding of the BCG process that has been developed in this study can be applied to the design of graphite materials. Control of flake size was previously demonstrated^8^ and it is clear from the present report that the morphology of the agglomerates of these flakes can also be controlled. These two morphological characteristics of graphite critically effect on the properties of the material and thus its performance in numerous applications, including as the active material in Li-ion battery anodes.

The conceptually simplest method to making BCG for Li-ion battery anodes is to graphitize biomass sources that have an appropriate particulate size range with appropriately sized catalyst particles. Sawdust is a readily available material and with a porous structure, that if preserved, could provide galleries that can mitigate the expansion of Si or other alloying active materials. However, its particle size dispersion is generally too large. Thus, as received sawdust was passed through stainless steel sieves, collected in five size distributions, charred and graphitized. Consistent with results shown previously in this report, the morphology of the graphite is largely identical to its sawdust precursor (Fig S7), typically splinter-like irregular rods or slivers, albeit significantly smaller due to shrinkage during charring. However, while splinter-like morphology from sawdust to graphite is retained as the sieving size is reduced, the aspect ratios of the graphite is less than those of the parent sawdust, particularly for the smallest graphite sample (− 400 mesh), presumably due to fracture during handling and hand grinding performed to break up the sample to aid Fe removal during purification.

The sawdust sieving fractions and the resulting graphite surface areas appear in Table 1. As expected, as the biomass size decreases the resulting graphite surface area generally increases. Higher surface area leads to an increase in Coulombic losses as the surface is passivated by the growth of a solid-electrolyte interphase (SEI) layer during Li-ion battery cycling. This effect is most prominent during the first cycle when the SEI is initially formed, and generally characterized by CE. As expected, the 1st cycle CE of the highest surface area fractions is significantly less than those with low surface areas (Table 1).

**Table 1:** Sawdust sizes after sieving, the resulting graphite surface area and the 1st cycle gravimetric capacity and CE of Li-ion anodes made from the BCG samples.

Sieve size (mesh)	Size range (micron)	Graphite surface area (m^2^/g)	1st cycle reversible capacity (mAh/g)	1st cycle CE (%)
+ 100	> 149	4.9	360	89.6
100–140	105–149	6	356	90.7
140–230	63–105	5.5	356	92.3
230–400	37–63	7.4	356	88.6
−400	< 37	14.2	335	83.2

BCG made from the three largest sized fractions had the lowest surface areas, falling within the range reported for commercial Li-ion graphites^31^. The largest fraction consisted of graphite slivers of 300–500 microns in length and 100–150 microns in width, much larger than dimensions of typical commercial Li-ion graphite (20–30 µm). BCG with dimensions that are similar to commercial graphite are not obtained in the largest two fractions, but that made from 140 to 230 mesh sawdust consist of slivers whose widths are primarily 10–30 µm, with a small fraction that are much larger (up to ~ 130 µm).

**Figure 6:**
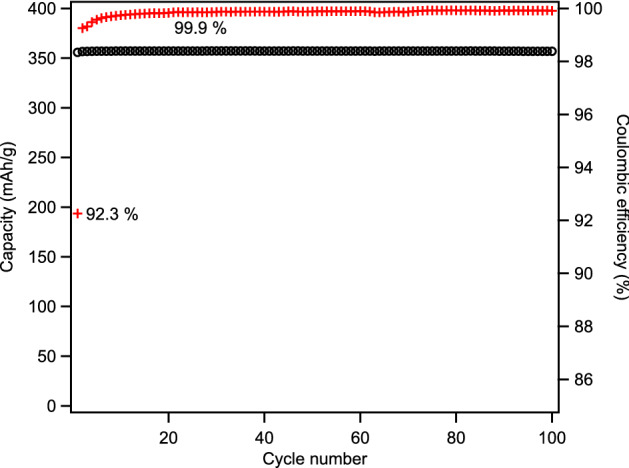
Capacity (black circles) and Coulombic efficiency (red crosses) of BCG made from 230 to 140 mesh sawdust.

BCG anodes made from 140 to 230 mesh sawdust exhibits the highest 1st cycle CE of the fractions, 92.3%, good capacity, 356 mAh/g rising to 357 mAh/g after one cycle and remaining constant through 100 cycles (Fig. 6). The performance of the largest fraction (+ 100 mesh) is very similar (Fig S9), as are the other fractions (Fig S10), but its 1st cycle CE is notably less despite having a lower surface area (Table 1). This may be due to poorer structural integrity; making uniform electrodes with very large grain graphite is difficult. Loss of electrical contact with a small fraction of the grains during charging and consequent expansion could result in irreversible losses due to stranding Li in those grains, lowering observed CE.

While the performance of BCG anodes made with selected sieved fractions of sawdust can be comparable to commercial graphite in capacity and CE, the macroporosity of the material limits volumetric capacity. However, this may prove favorable as a host in a composite anode with alloying metals (e.g. Si). The retention of the morphology of the biomass resulted in a structure comprised of rigid graphite walls surrounding macropores that could provide galleries for alloying metal expansion. While the porosity of this graphite may or may not be ideal for alloy inclusion, the tremendous variety of biomass structures available, and the retention of biomass structure when transformed to graphite, provides ample opportunity for the rational selection of host graphites for such a purpose.

### Spherical by design

The average size of commercial Li-ion anode shaped graphite agglomerates, presumably measured in their longest dimension, has been reported to range from 8 to 30 µm^32^. In an attempt to create BCG engineered to mimic the morphology and performance of commercial graphite, cellulose spheroids were chosen as the starting biomass material. Accounting for the expected mass loss during charring/graphitization, nominally 30 µm diameter cellulose was chosen to yield graphite spheroids of appropriate size. Examination with SEM found that the morphology of the spheroids is somewhat irregular, with smooth albeit moderately pitted surfaces (Fig. S11). The average and median largest dimension of the spheroids was determined to be 17.9 and 16.0 µm respectively. Although most are ~ 15 to 30 µm in their largest dimension, a significant portion were much smaller, some small than 10 µm (Fig S12). A small number were found to have largest dimensions that exceeded 30 µm, however, these were distinctly prolate with an equatorial diameter of less than 30 µm. While it is clear that the size distribution could be readily reduced through sieving or other selection techniques, ultimately affecting BCG properties, no such attempts were made for this preliminary study.

**Figure 7:**
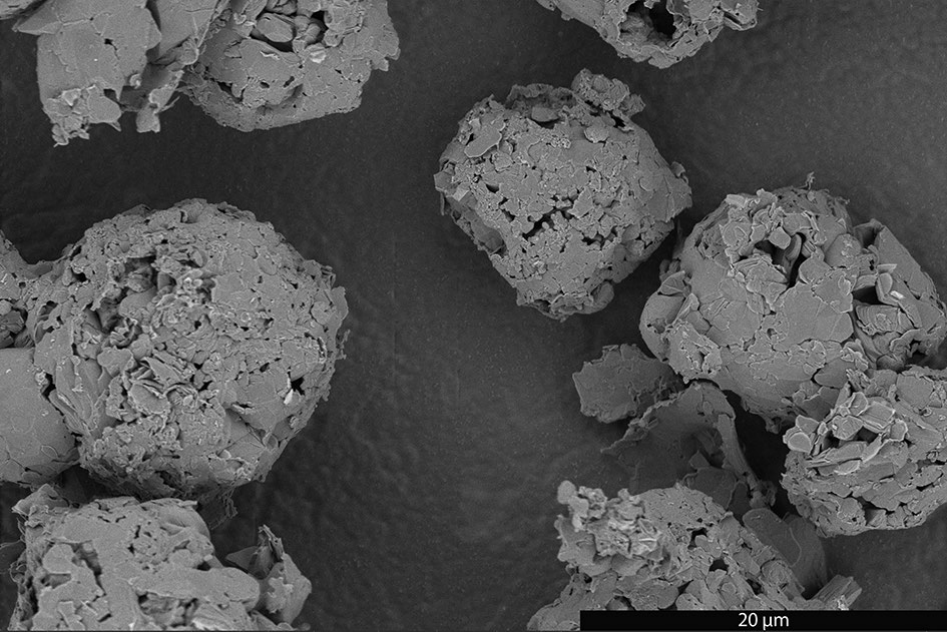
SEM image of BCG made from cellulose spheroid char.

Charring did not change the general appearance of the spheroids; they retained an irregular spheroidal shape and smooth, moderately pitted surfaces (Fig S13). Their size, however, was clearly reduced with an average and median largest dimension of 12.9 and 13.7 µm respectively (Fig S14). The spheroidal shape was largely retained after graphitization, but the surface texture was dramatically altered. The surface of the spheroidal BCG (sBCG) is predominately composed of the basal planes of graphite flakes connected in a “jigsaw puzzle” like manner with some gaps (Fig. 7, Fig S15). The average and median largest dimension of the sBCG was found to be 15.2 and 15.1 µm, respectively, with ~ 75% between 13 and 20 µm (Fig S16). However, the tendency of the BCG spheroids to adhere to one another made dispersion of the material difficult, lowering confidence that the quantitative measurements of the size distribution were representative. The size distribution for the sBCG is very similar to that of the spheroid char, with the exception of the < 8 µm size range. It seems probable that smallest sBCG agglomerates were obscured by close interaction with larger agglomerates, or perhaps selectively lost during purification, resulting in their undercounting.

For comparison to sBCG, SEM images were obtained of commercial Li-ion battery graphite, Hitachi MagE3. MagE3 consists of irregularly shaped agglomerates of flake graphite, some of which are roughly spheroidal with all, or nearly all, having rounded edges. Their size distribution ranges from less than 3 to more than 60 µm in their largest dimension (Figs. S17, S18). The largest fraction, more than 50% of the agglomerates, appears to be less than 30 µm with ~ 20 to 30% of them being less than 10 µm, and some agglomerations of a few or single loose flakes present. The expansive breadth of the sizes and the irregularity of the shapes made quantitative measurements of the distribution from these SEM observations too fraught with potential error to present. However, the observed distribution is in reasonable agreement with the previously reported^33^ average size (d-50) of 22.4 µm and a published SEM image^31^.

Morphologically, sBCG and MagE3 are similar in that they are composed of flake graphite agglomerates with rounded edges. The shapes of the sBCG agglomerates are much more regular, dramatically closer to true spheres. The median size of the sBCG is significantly smaller and their size distribution is much narrower. The median volume of the sBCG agglomerates is ~ 24 to 33% of that of MagE3, depending on whether the char or measured sBCG distribution is more representative of the true sBCG distribution, calculated as ideal spheres.

**Figure 8:**
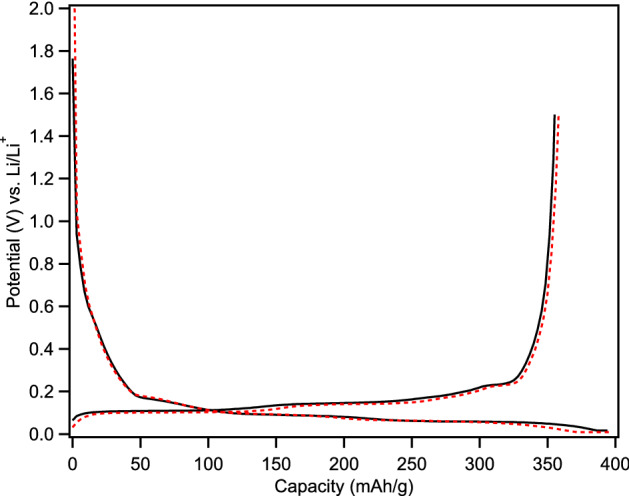
First cycle galvanic charge and discharge curves of sBCG (solid black lines) and MagE3 (dashed red line).

**Figure 9:**
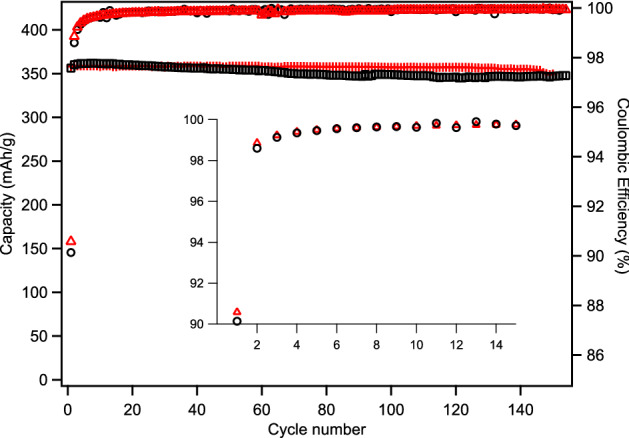
Capacity (left axis) and Coulombic efficiency (right axis) of MagE3 (red crosses and triangles, respectively) and sBCG (open black squares and circles, respecively) plotted as a function of charge/discharge cycles. Inset is an expanded plot of Coulombic efficiency of the first 15 cycles with scale on left axis.

Despite the seemingly much larger size of MagE3, its surface area is only 8.1% smaller (3.08 vs 2.83 m^2^/g). This can also be seen in the first cycle CE of anodes made with MagE3 and sBCG, where the former (90.6%) is only marginally higher than the later (90.2%). In fact, the first cycle performance is remarkably similar, with nearly identical galvanic charge and discharge curves (Fig. 8), and MagE3 attaining 0.8% higher first cycle capacity (358 vs 355 mAh/g). The capacity retention was nearly identical, with MagE3 obtaining an average of 1% better capacity, finishing 150 cycles with 0.8% higher capacity (Fig. 9 and Fig S19).

## Conclusions

Herein, morphological control of graphite synthesized from biomass by a carbon negative method has been demonstrated. Whereas commercial graphite production and shaping is highly deleterious to the environment and requires facilities that are generally unobtainable to the research laboratories, shaping for the BCG process is environmentally benign and can be done with malleable biomass and graphitization with readily obtainable equipment. The BCG method allows for an unprecedented level of rational morphological and flake size control at the laboratory scale. This capability may be of particular importance to Si/graphite composite anode research, allowing the rational design of optimal graphite porosity to accommodate expansion.

Graphite basal planes invariably dominate the exterior surfaces of the agglomerates produced by the BCG method. This feature is fortuitous for application in Li-ion anodes as it minimizes the exposure of the edge site to the electrolye in a cell. Edge sites form thicker SEI layers than basal planes because they are more reactive undergo an expansion/contraction cycle during lithiation/delithiation that leads to SEI fracture and reformation^34^. Commercial graphite achieves a similarly basal plane dominated surface form as a result of mechanical shaping, whereas it occurs in BCG as a consequence of the graphitization mechanism.

It should be noted that the material produced for this study was done so as a preliminary exploration of the size and shape control made possible by the BCG method, not to produce optimized Li-ion anode graphite. It is unclear how sBCG, or any of the BCG made to date, would perform in anodes made by commercial production methods. Further studies would be required to evaluate the volumetric density, cycle life, rate capability, calendar life and other performance characteristics one might achieve with BCG. However, the level of control enabled by the method should allow for rapid optimization. Truly narrow size and shape distributions that are not currently possible with commercial shaping are readily accessible by the BCG method. Narrow size distributions could allow the development of electrodes with uniform pores, the graphite agglomerates acting as “scaffolding” supporting and directing the structure much like, although undoubtedly with less precision than, the larger of the ions in an ionic compound. This would create open galleries, customizable by changing the graphite agglomerate size and shape, and by formulating graphite mixtures of different sizes and shapes. So, rather than using commercial graphite, optimized to form electrodes with minimal porosity, one could design the graphite to produce optimal pore shape and size for alloying materials. The rigid galleries could provide space for alloying metals to expand and contract without local or global electrode expansion that can led to mechanical degradation. In addition, it is not clear that the currently used commercial “potato” or spheroidal morphology is optimal or merely the best that can achieved given the limitations of industrial graphite shaping; while it is even less clear what morphology might perform better, if one could be found (e.g. by simulation), and the method to achieve that shape in biomass can be developed, it seems highly likely that the shape can be retained during BCG graphitization.

## Methods

### Materials

All materials were used as received unless otherwise noted. Unless stated otherwise in this manuscript, hardwood sawdust (CrossRoad Sales LLC) and < 10 µm Fe metal (Alfa Aesar iron powder, 99.5%, product no. 00170A1, Fig S1), were used as the starting materials. In addition, < 210 µm Fe (Acros, 99%, −70 mesh, product no. 197815000) was used after sieving to 200–170 mesh (74–88 µm) and referred to as “ ~ 80 µm Fe”. Other biomass used includes algae (Anthony’s Organic Chlorella Powder) and cellulose spheroids (Cellulobeads D-30, Kobo Products, Inc.).

### Synthesis of graphite

The procedure followed here is largely the same as previously published, with the notable exception of initiating the transformation of bio-char to graphite with a diode, rather than a CO_2_ laser^8^. Typically, biomass (6.0 g) and Fe metal (2.0 g) were mixed using a ball mill and pressed to form 20 mm diameter pellets. A 21/64’’ hole was then drilled in the center of each pellet, and the pellets were subjected to pyrolysis under N_2_ gas (600 °C). The heating under inert atmosphere resulted in the evolution of bio-oil and gas and transformed the biomass to biochar. After charring at 600 °C, 40% of the original pellet mass remained (80% of sawdust mass lost) as black pellets containing biochar (37.5 wt %) and Fe (62.5 wt %). After they were cooled, the biochar/Fe pellets were skewered on a ¼’’ diameter stainless steel rod and irradiated by a 2 mm diameter 980 nm laser beam (200 W, BWT Beijing) while being rotated in an evacuated (0.5 Torr, with flowing inert gas) chamber. Purification was performed in a manner identical to that previously published.

### Powder X-ray diffraction (XRD)

XRD patterns were obtained with a Bruker D2 Phaser X-ray diffractometer using Cu Kα radiation and a LYNXEYE XE-T detector.

### Surface area determination

Surface area (BET method) was determined using from nitrogen adsorption isotherms obtained with a Tri-Star 3000 (Micrometrics) in the same manner as previously published^8^. One of the three sample measurement ports of the Tri-Star was equipped with an empty sample tube with which the saturation vapor pressure (P_0_) of N_2_ was measured concurrently with each measurement of the equilibrium vapor pressure (P) over the sample.

### Electron microscopy

Scanning electron microscopy (SEM) micrographs were obtained using a FEI Teneo LV with its in-lens secondary electron detector using 1.00 and 2.00 kV accelerating voltage. Transmission electron microscopy (TEM) micrographs were obtained using a FEI Talos F200X TEM operating at 200 kV and a Ceta 16 M camera.

### Electrode preparation and cycling

Electrochemical testing was similar to that done for a previously published study, with minor modifications to the formulation procedures^8^. A graphite anode was formulated by combining 270 mg of graphite (BCG or MagE3), 15 mg of carbon black (Super C45, Imerys TIMCAL America Inc.), and 300 μL of 5% Li-polyacrylate binder solution prepared by dissolving poly(acrylic acid) (1000 kDa, Polysciences) in deionized water and neutralizing with LiOH (95%, Strem). Mixing was performed using a Mazerustar planetary mixer in a 6 mL polypropylene container (Yamayu) equipped with 3 stainless steel balls (5 mm diameter) at 1000 RPM for 10 min to form a slurry. Final adjustments to the slurry viscosity were made by adding small quantities (< 100 μL) of water and mixing for an additional 10 min at 100 RPM. The slurry was cast onto copper foil (0.127 mm, 99.9%, Alfa Aesar) and dried under vacuum at 150 °C for 2 h. Round electrodes (16 mm diameter) were cut from the resulting sheet using a die cutting press (MSK-T-07 Precision Disc Cutter, MTI Inc.).

Coin cells (CR2016, MTI Inc.) containing the BCG anode and Li metal (99.9%, MTI Inc.) electrodes separated by a polypropylene porous membrane (Celgard 3401) were assembled in an Ar filled dry box (< 0.1 ppm O_2_ and H_2_O). The electrolyte used was 1 M LiPF_6_ in EC:DMC (1:1 v/v) mixture (battery grade, < 15 ppm H_2_O content, Sigma Aldrich) with 10% FEC (> 99%, Solvay) by volume. The choice to use FEC additive was made due to its prevalence of its use in Si/graphite composite anode studies. Electrochemical cycling was performed using an Arbin Instruments BT2000 Battery Test System. The cell was discharged (Li loading into BCG) at constant current (C/2) from open circuit voltage to a cutoff potential of 10 mV Li/Li^+^ and charged (Li unloaded from BCG) at the same current to 1.5 V vs. Li/Li^+^. The cells were rested for 15 min between discharge and charge cycles.

## Supplementary Information

Supplementary Figures.

## Data Availability

The datasets used and/or analysed during the current study are available from the corresponding author upon reasonable request.
