# Supplementary material for: Sustainable conversion of biomass to rationally designed lithium-ion battery graphite
Source: Sci Rep. 2022 May 16;12:8080. doi: 10.1038/s41598-022-11853-x (PMC9110727; doi:10.1038/s41598-022-11853-x)
Supplement: Supplementary file 1 — Supplementary Figures. [file 41598_2022_11853_MOESM1_ESM.pdf]

Supporting Information

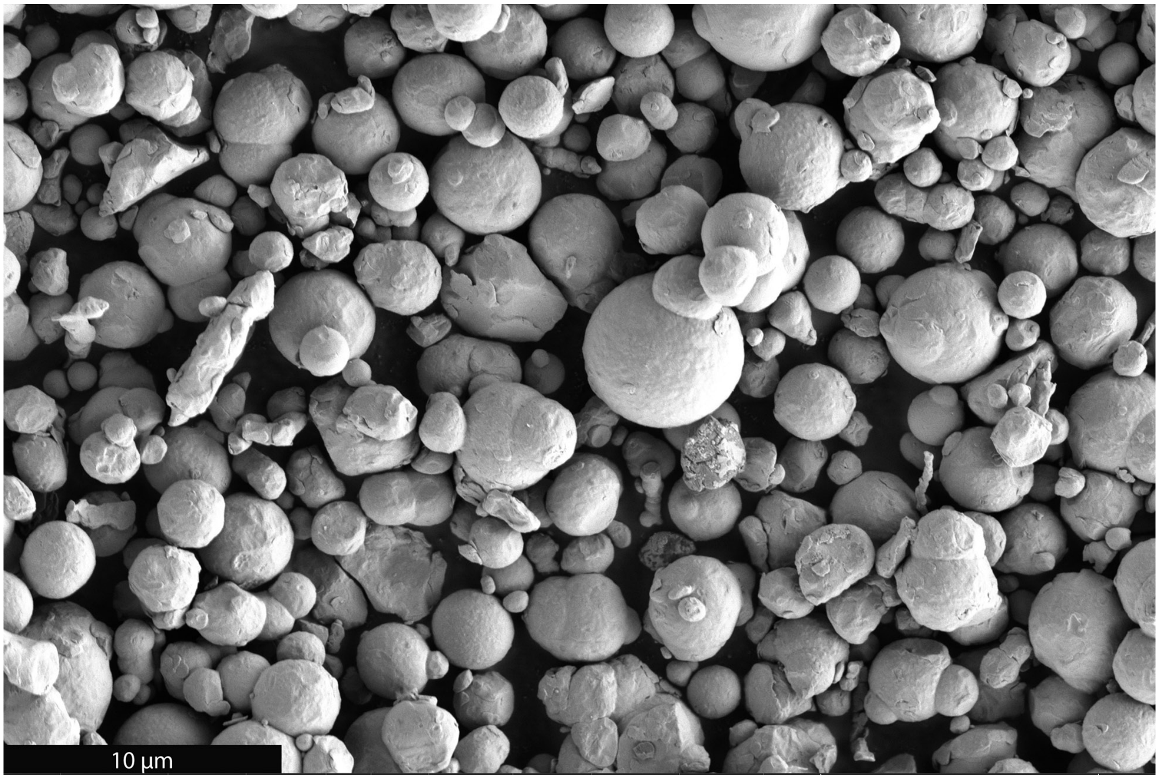

Fig S1 – SEM image of < 10 μm Fe use for BCG synthesis.

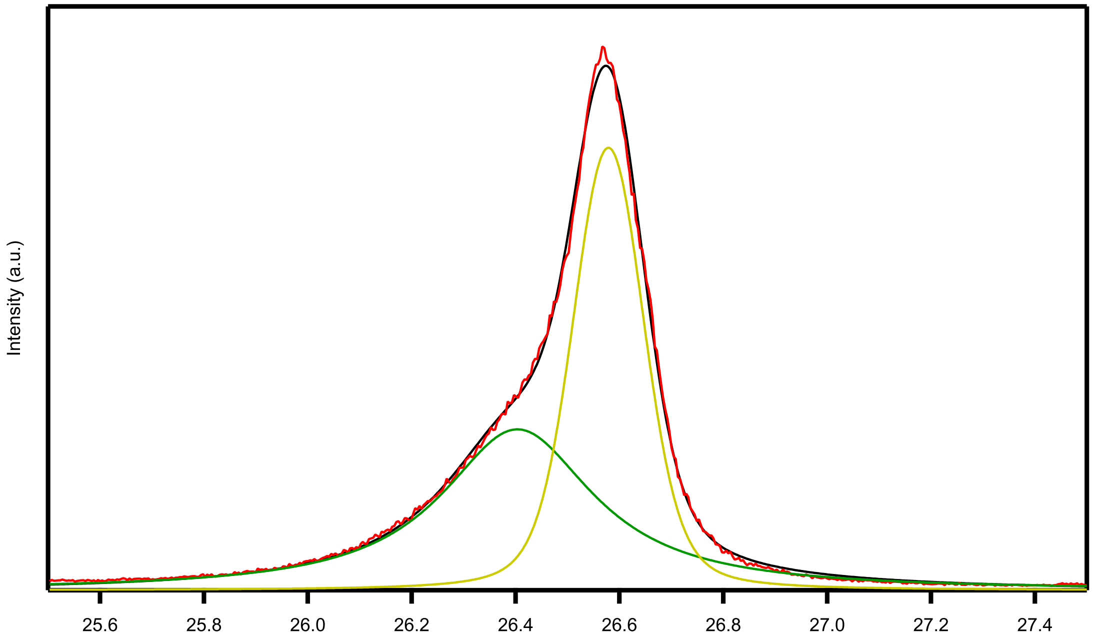

Fig S2 – Powder XRD pattern of the product of 15 W irradiation during a 240 s rotation (red) and fit of the data (black) by deconvolution into two peaks (yellow and green).

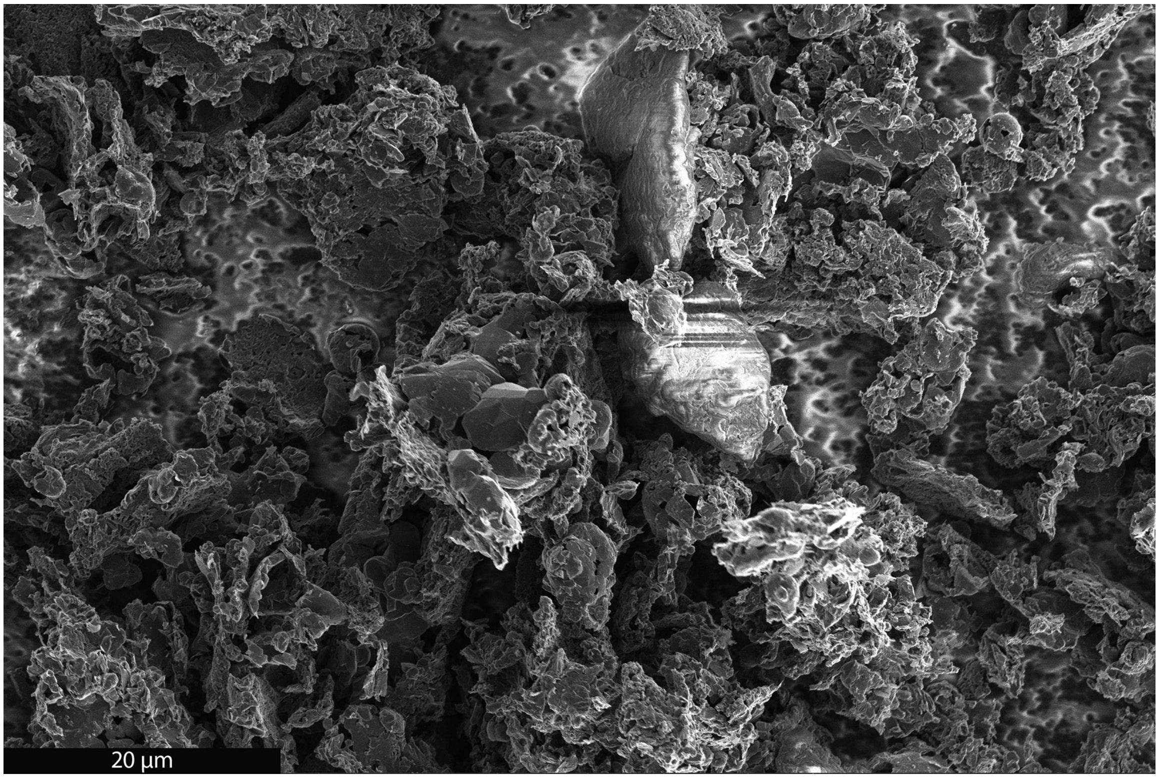

Fig S3 – SEM image of the product of 15 W irradiation during a 240 s rotation showing what appears to be some small graphite flakes (center left) among poorly crystalline carbonaceous material.

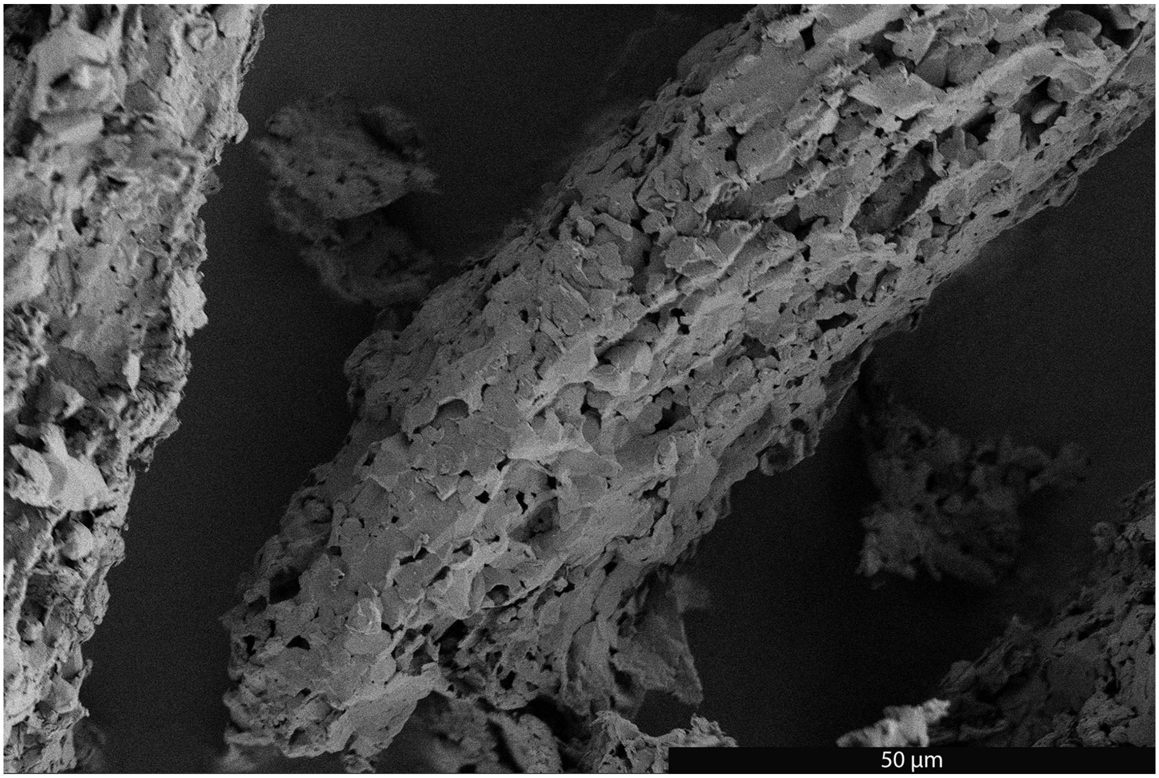

Fig S4 – SEM image of BCG made from sawdust showing that the surface is primarily composed of the basal planes of interconnected graphite flakes.

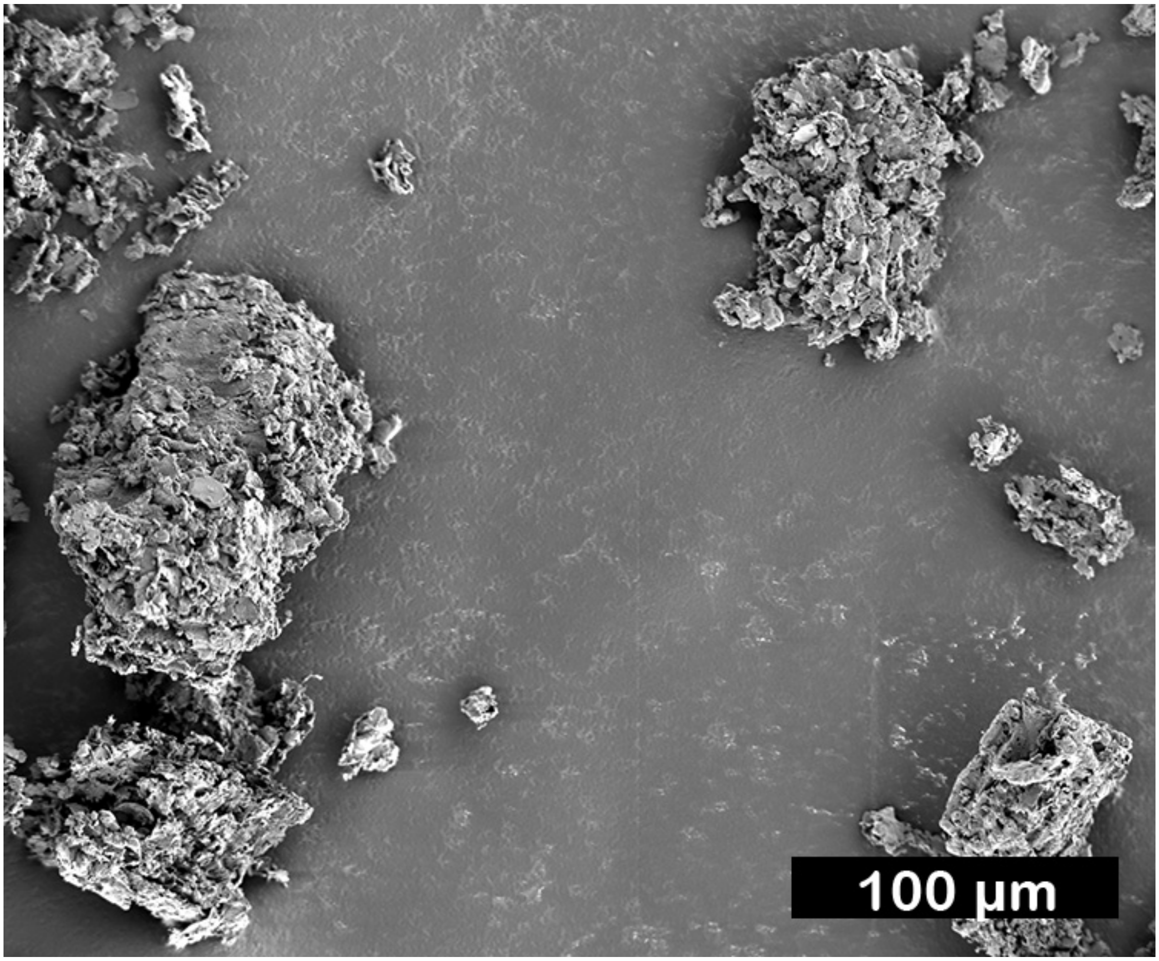

Fig S5 – SEM of graphite “potato” agglomerates made from materials mixed in a ball mill for 30 min.

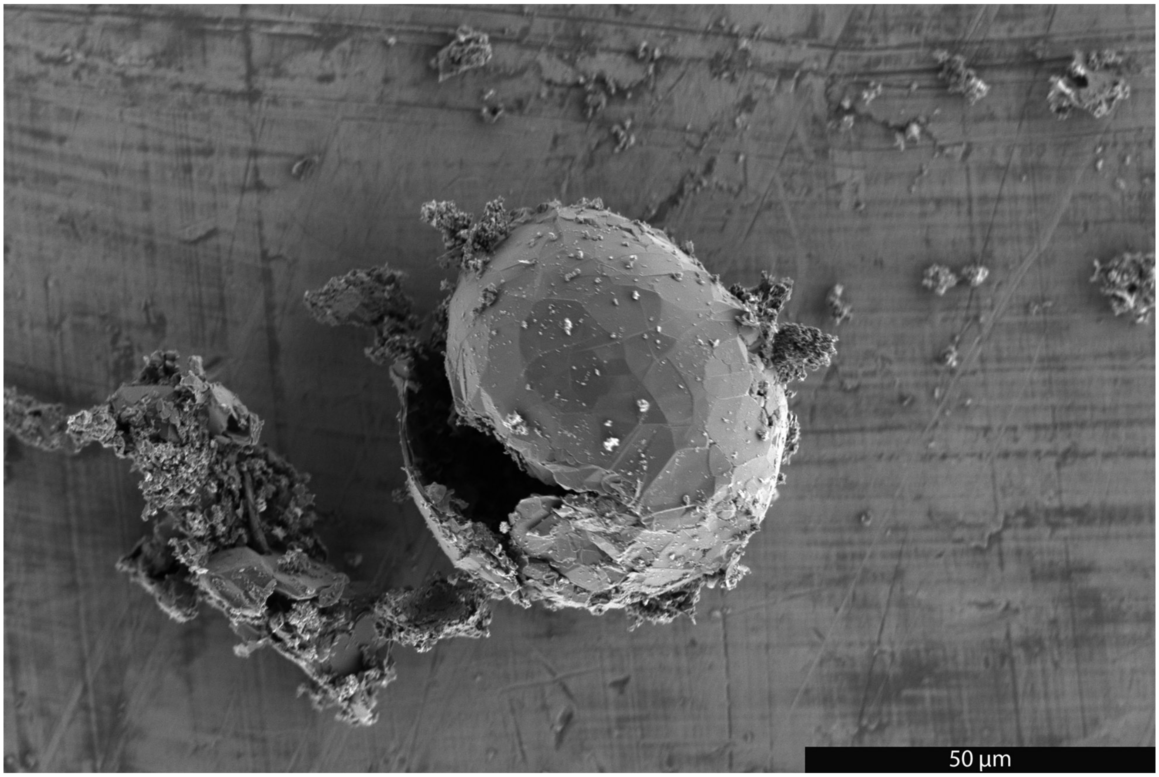

Fig S6 – SEM image of broken BCG sphere made from spherical algae showing that the graphite flakes that make up the exterior are very thin (< 1 µm).

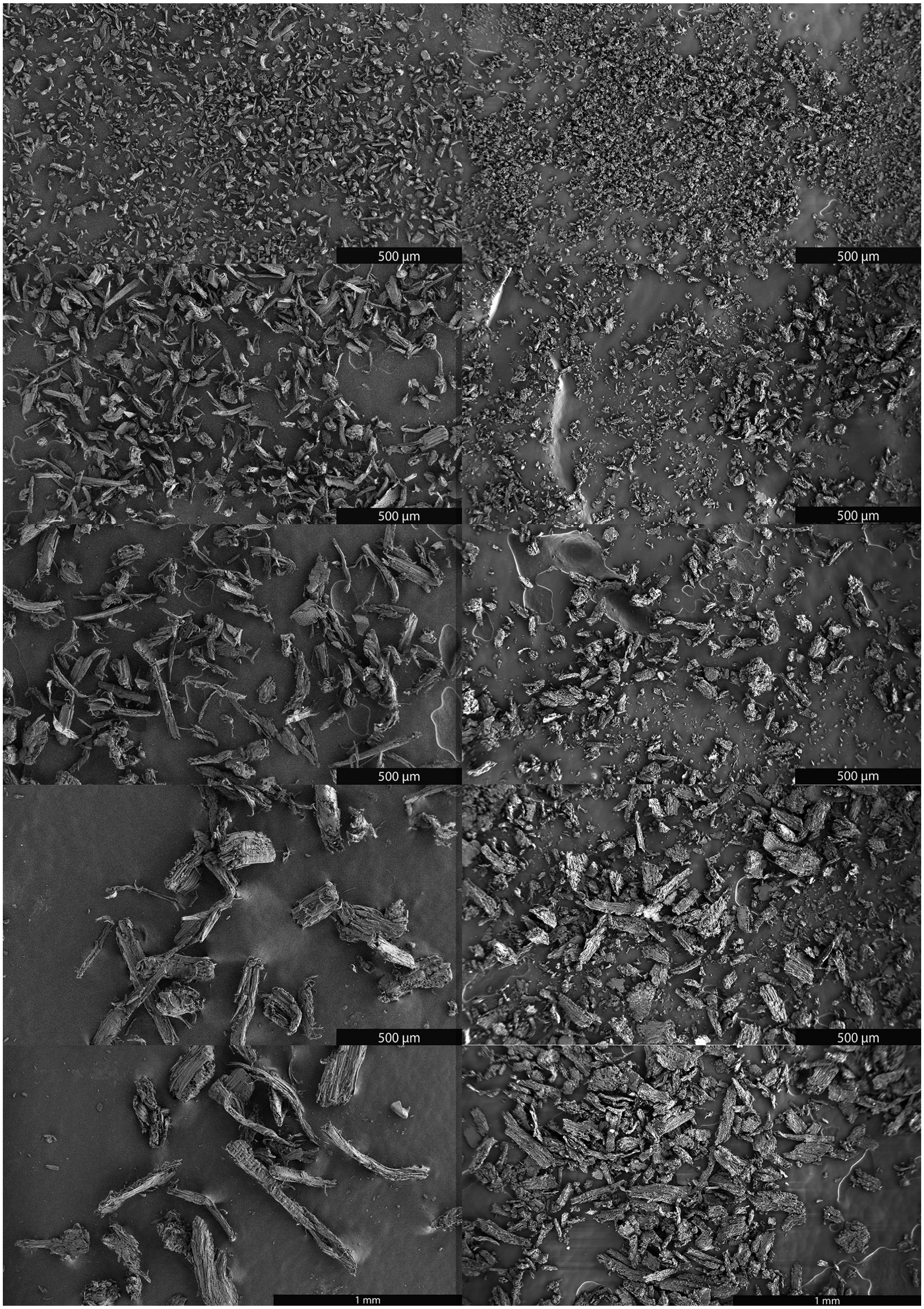

Fig S7 – SEM images of sawdust (left) and corresponding resulting BCG (right) made from sawdust size selected by sieving. The sawdust size fractions are -400, 400 – 230, 230 – 140 and +100 mesh from top to bottom respectively.

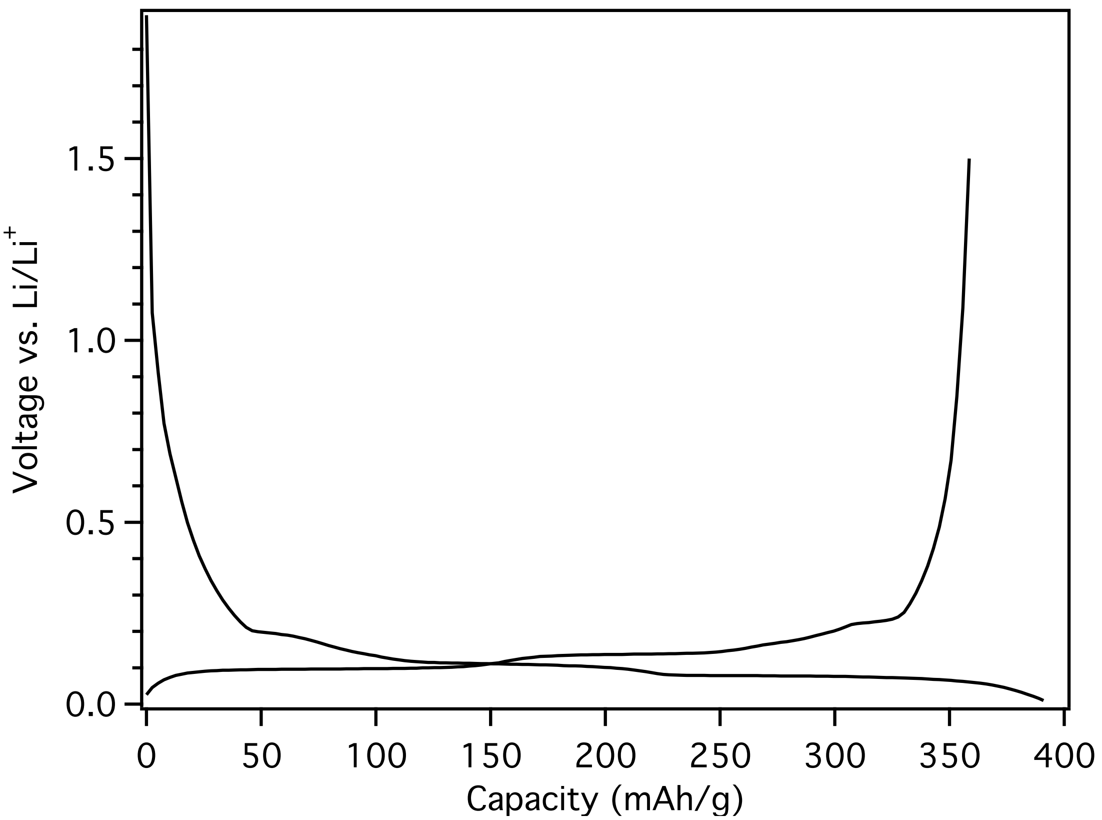

Fig S8 – First cycle galvanic charge and discharge curves of BCG made from 230 – 140 mesh sawdust.

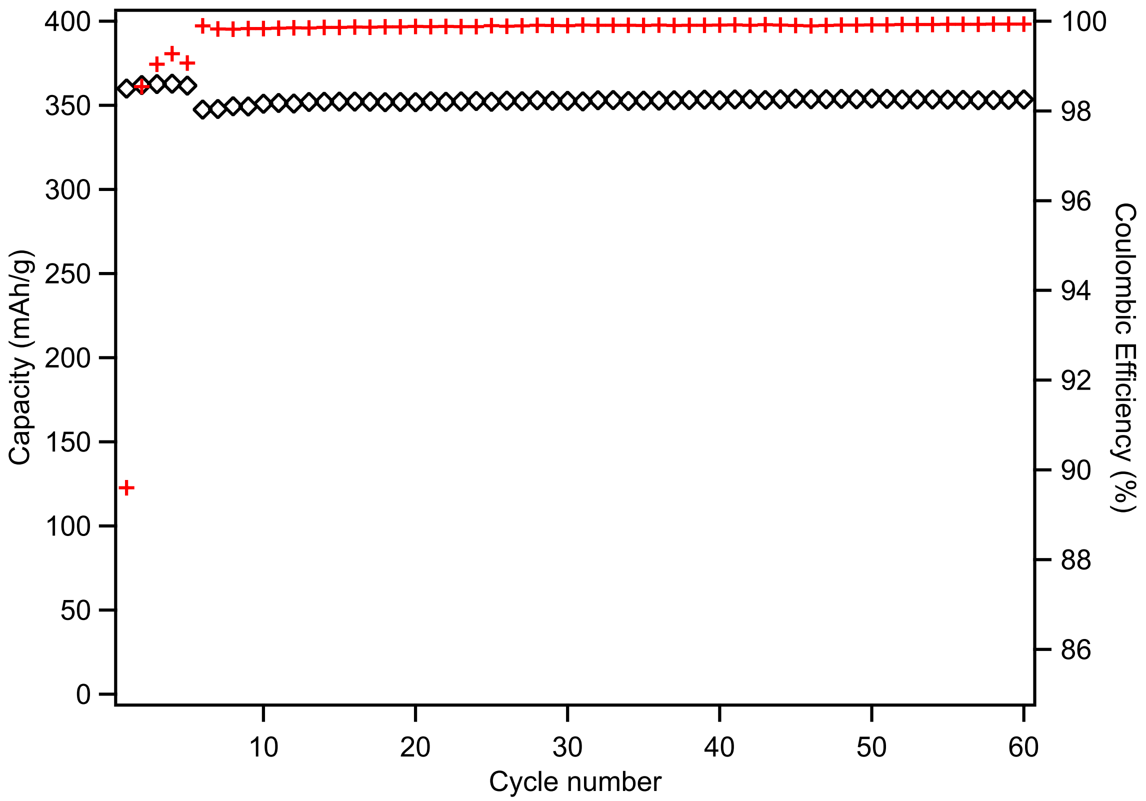

Fig S9 – Capacity (black diamonds) and Coulombic efficiency (red crosses) of BCG made from +100 mesh sawdust cycled at C/20 during the first 5 cycles and then C/4 for the remaining cycles.

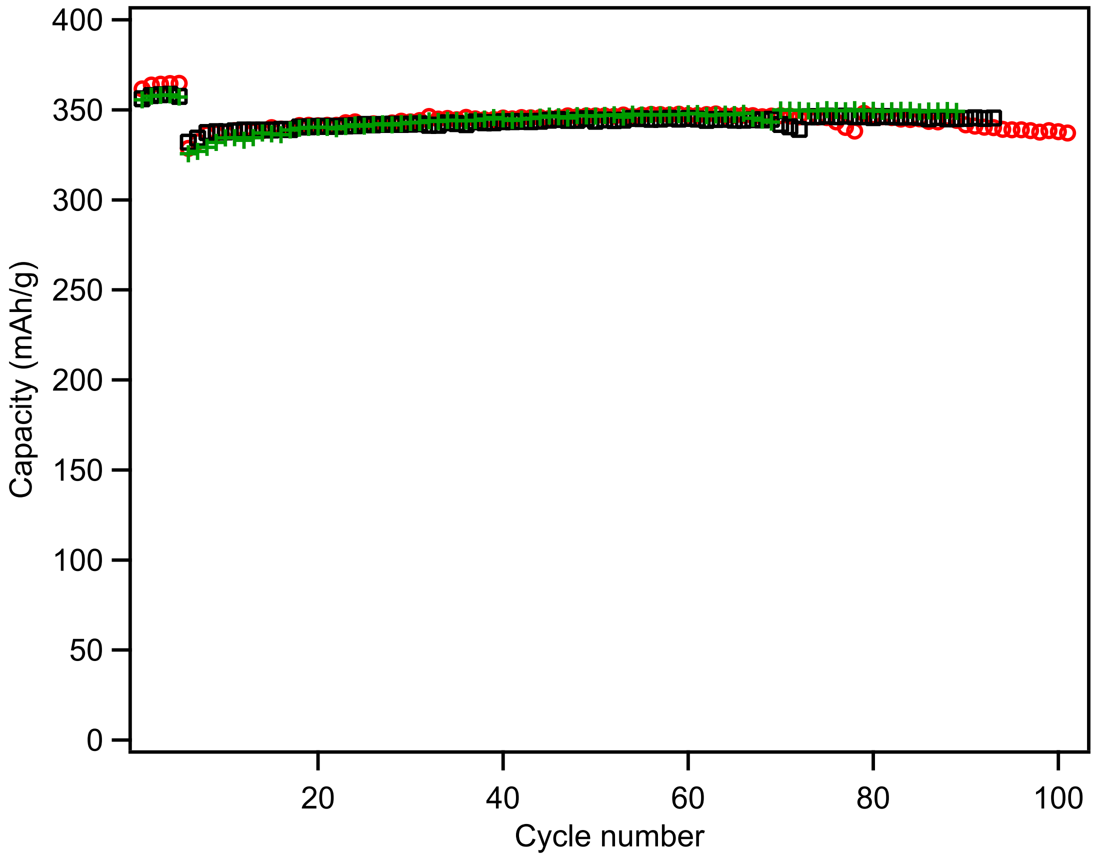

Fig S10 – Capacity of BCG made from 100 – 140 (green crosses), 230 – 400 (black squares) and – 400 (red circles) mesh sawdust cycled at C/20 during the first 5 cycles and then C/4 for the remaining cycles.

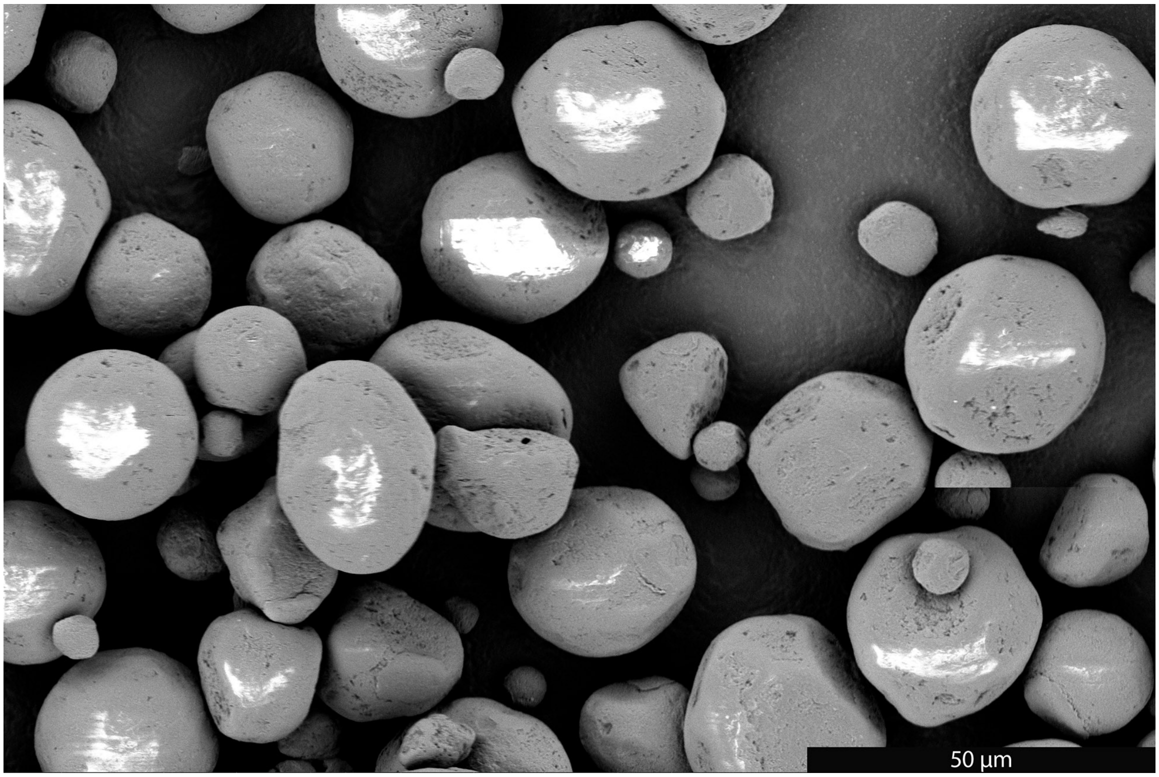

Fig S11 – SEM image of cellulose spheroids.

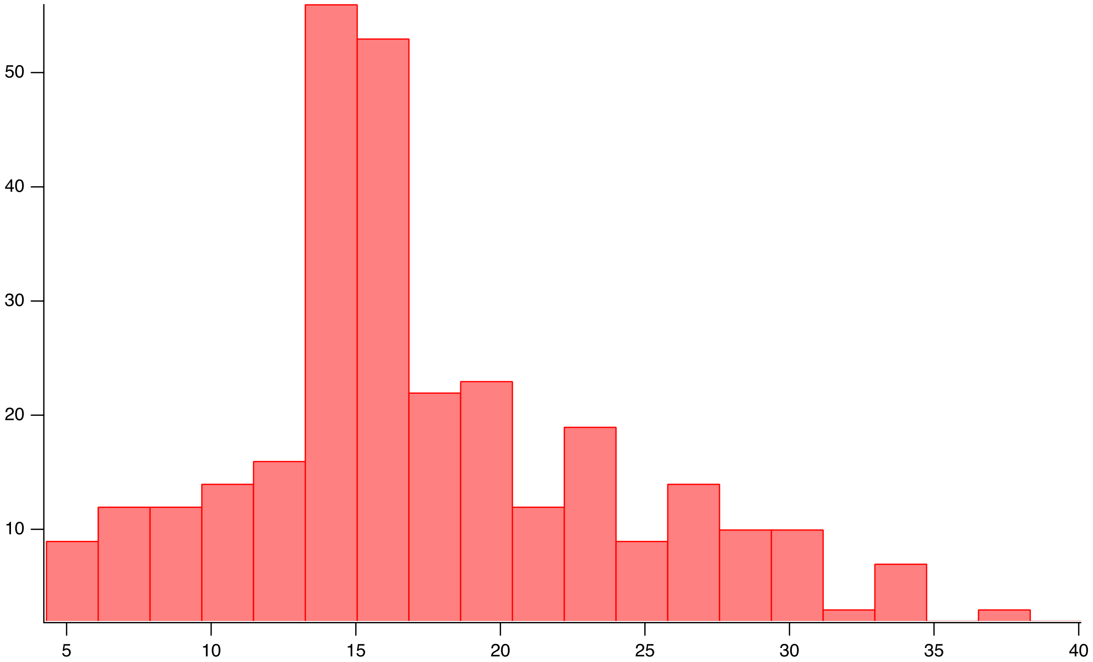

Fig S12 – Size distribution of the cellulose spheroids.

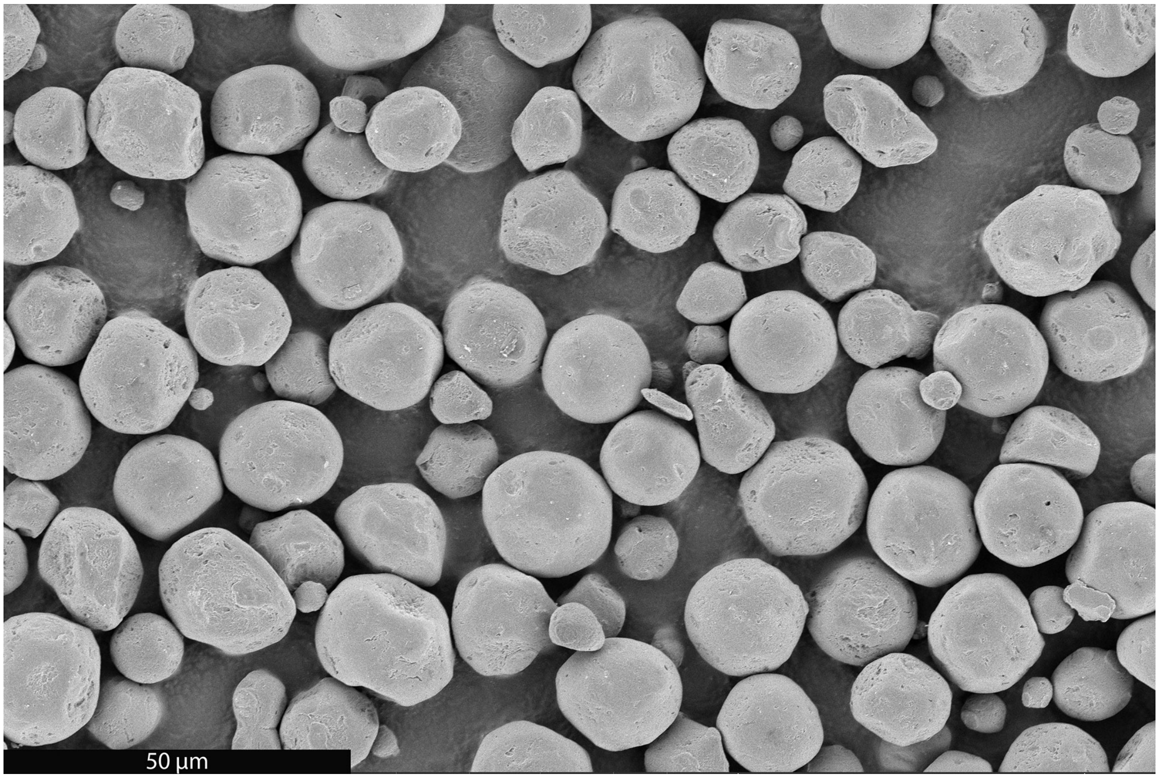

Fig S13 – SEM image of cellulose spheroid char.

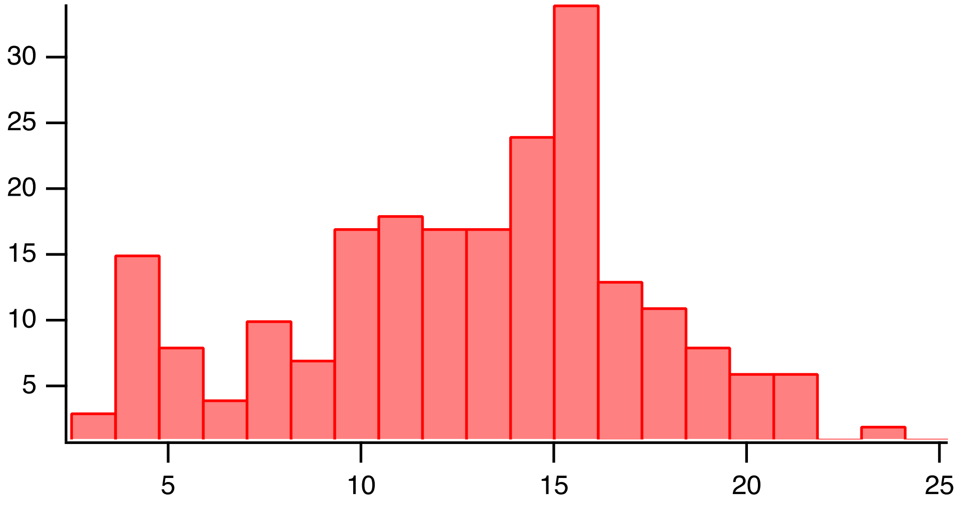

Fig S14 - Size distribution of the cellulose spheroid char.

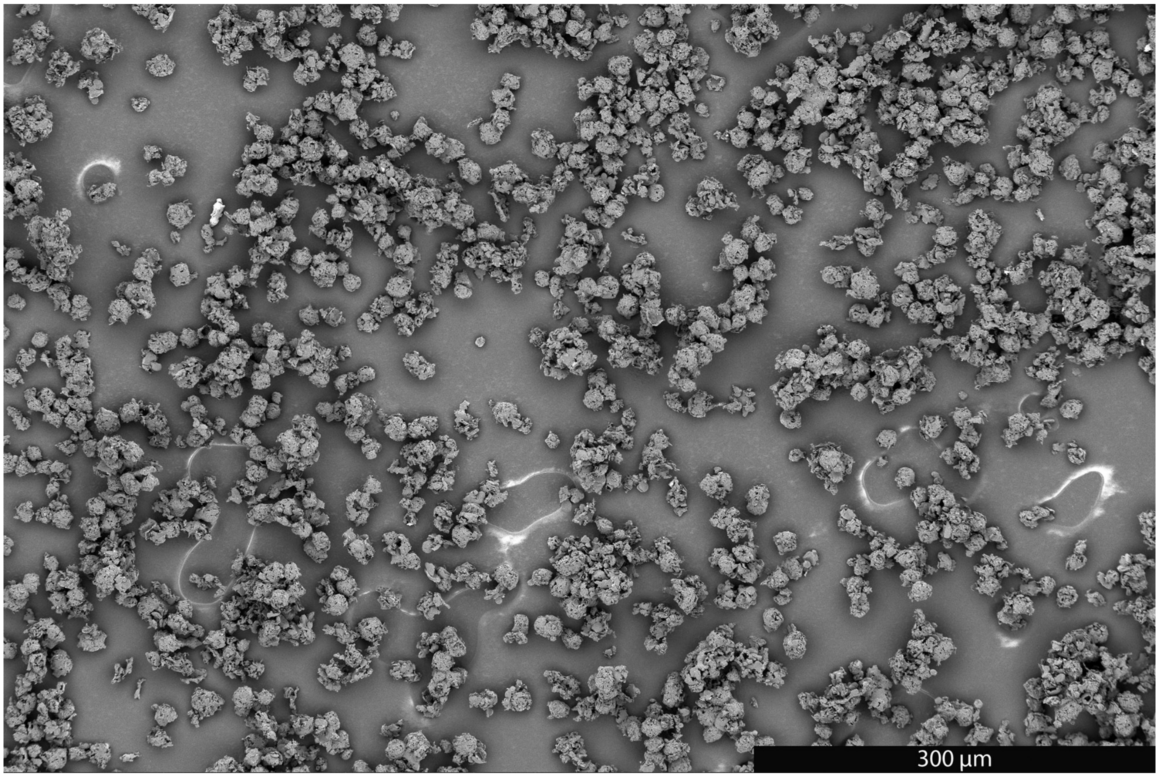

Fig S15 – SEM image of BCG made from cellulose spheroid char.

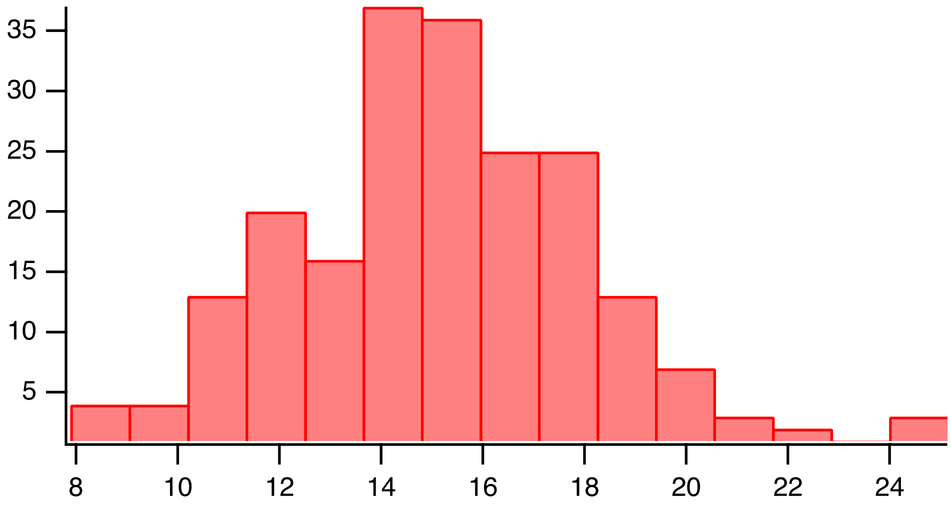

Fig S16 - Size distribution of the BCG made from cellulose spheroid char.

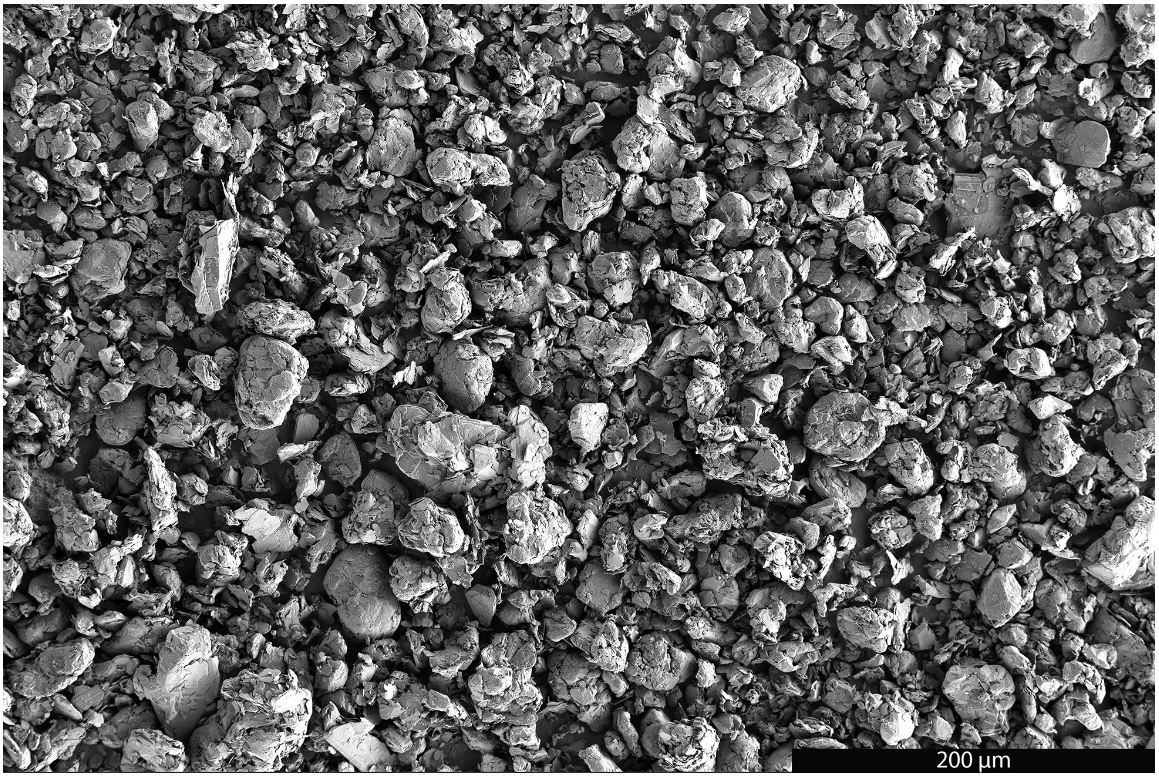

Fig S17 – SEM image of Hitachi Mage3 commercial Li-ion battery anode graphite.

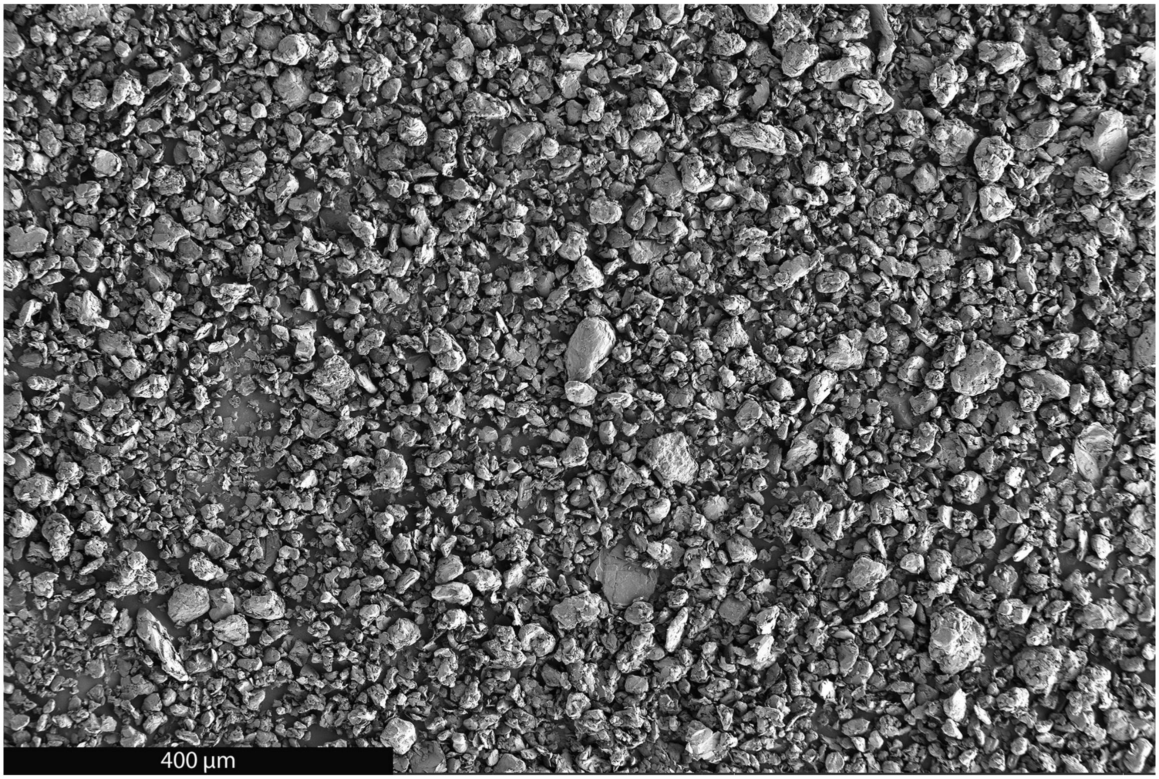

Fig S18 – SEM image of Hitachi Mage3 commercial Li-ion battery anode graphite.

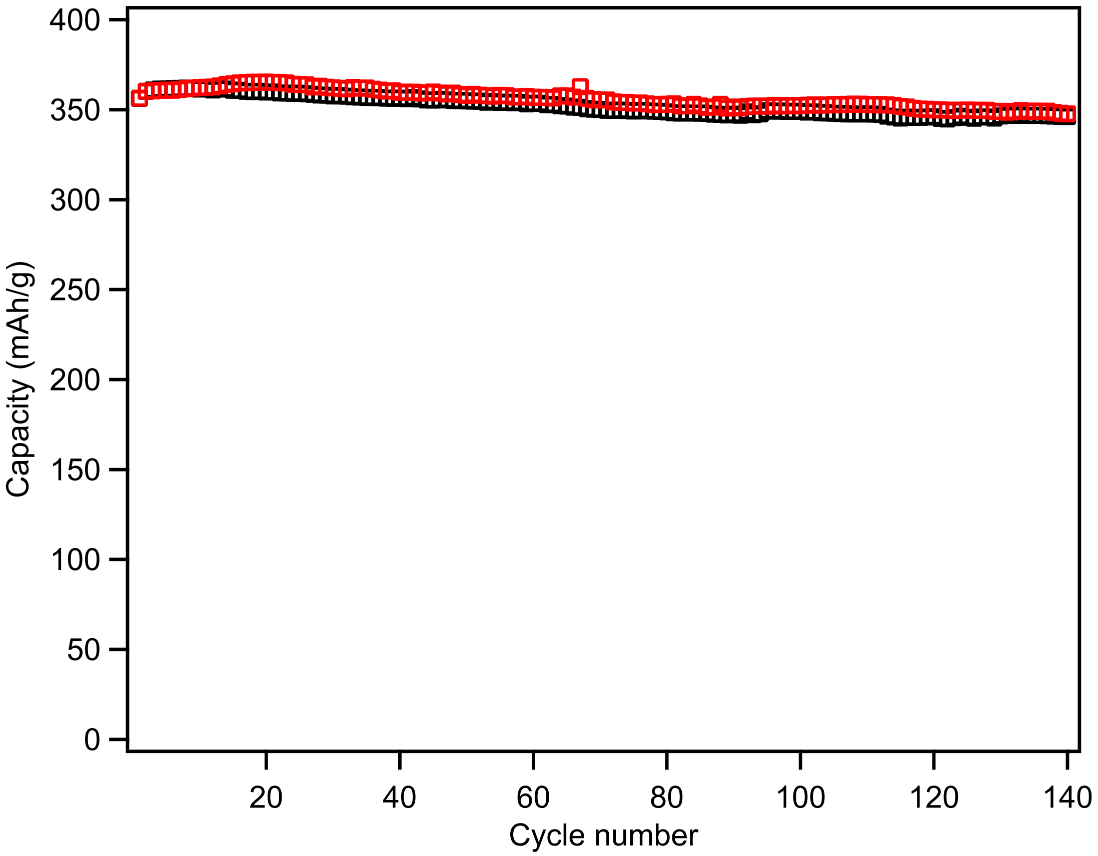

Fig S19 – Capacity of replicant sBCG electrodes plotted as a function of charge/discharge cycles.
